# Preparation and Characterization of Polymeric Microparticles Based on Poly(ethylene brassylate-co-squaric Acid) Loaded with Norfloxacin

**DOI:** 10.3390/pharmaceutics16040550

**Published:** 2024-04-17

**Authors:** Alexandru-Mihail Șerban, Isabella Nacu, Irina Rosca, Alina Ghilan, Alina Gabriela Rusu, Loredana Elena Niță, Raluca Nicoleta Darie-Niță, Aurica P. Chiriac

**Affiliations:** 1 Department of Natural Polymers, Bioactive and Biocompatible Materials, “Petru Poni” Institute of Macromolecular Chemistry, 41-A Grigore Ghica Voda Alley, 700487 Iasi, Romania; serban.alexandru@icmpp.ro (A.-M.Ș.); cobzariu.isabella@gmail.com (I.N.); diaconu.alina@icmpp.ro (A.G.); rusu.alina@icmpp.ro (A.G.R.); achiriac@icmpp.ro (A.P.C.); 2 Biomedical Sciences Department, Faculty of Medical Bioengineering, Grigore T. Popa University of Medicine and Pharmacy of Iasi, 9-13 Kogalniceanu Street, 700454 Iasi, Romania; 3 Center of Advanced Research in Bionanoconjugates and Biopolymers, “Petru Poni” Institute of Macromolecular Chemistry, 41-A Grigore Ghica Voda Alley, 700487 Iasi, Romania; rosca.irina@icmpp.ro; 4 Physical Chemistry of Polymers Department, “Petru Poni” Institute of Macromolecular Chemistry, 41-A Grigore Ghica Voda Alley, 700487 Iasi, Romania

**Keywords:** polymeric microparticles, norfloxacin, oral administration, bio-based polyester

## Abstract

In recent years, increasing interest has been accorded to polyester-based polymer microstructures, driven by their promising potential as advanced drug delivery systems. This study presents the preparation and characterization of new polymeric microparticles based on poly(ethylene brassylate-co-squaric acid) loaded with norfloxacin, a broad-spectrum antibiotic. Polymacrolactone was synthesised in mild conditions through the emulsion polymerization of bio-based and renewable monomers, ethylene brassylate, and squaric acid. The microparticles were obtained using the precipitation technique and subsequently subjected to comprehensive characterization. The impact of the copolymer/drug ratio on various properties of the new system was systematically evaluated, confirming the structure of the copolymer and the encapsulation of norfloxacin. The microspheres are approximately spherical and predominantly homogeneously distributed. The average hydrodynamic diameter of the microparticles falls between 400 and 2000 nm, a decrease that is observed with the increase in norfloxacin content. All samples showed good encapsulation efficiency and drug loading capacity, with the highest values obtained for microparticles synthesised using an equal ratio of copolymer and drug. In vitro drug release results disclose that norfloxacin molecules are released in a sustained biphasic manner for up to 24 h. Antimicrobial activity was also studied, with samples showing very good activity against *E. coli* and moderate activity against *S. aureus* and *E. faecalis*. In addition, HDFA human fibroblast cell cultures demonstrated the cytocompatibility of the microparticles.

## 1. Introduction

Nowadays, the use of micro- and nanoparticles in the biomedical field, especially in the delivery and controlled release of therapeutic agents, has attracted increased attention due to their remarkable properties. Particle-based drug delivery systems present advantages compared to other systems due to their small dimensions, high surface-to-volume ratio, higher circulation time, improved antibacterial activity, and enhanced biocompatibility [1,2]. A variety of micro- and nanostructures, such as polymeric [3,4], lipidic [5,6], metallic [7,8], ceramic [9], or carbon-based [10,11] have been reported as promising drug delivery systems. Among various types of microparticles, those based on natural and synthetic polymers are particularly preferred in drug delivery applications due to their higher encapsulation capacity, biocompatibility, biodegradability, and improved cellular uptake. Chitosan-based composite nanoparticles with pH and temperature-triggered release were reported as potential delivery platforms for anti-inflammatory drugs, such as diclofenac [2]. In another study, collagen nanoparticles loaded with gentamicin synthesised by electrospraying were used as a coating biomaterial for microneedle patches [12]. Although natural polymers have gained extensive attention in the last decade, synthetic polymers like polyesters, polyanhydrides, polyorthoesters, or polydioxanones, remain the most used [13]. This is due to the controlled chemical composition, the reproducibility of batches, and their cost effectiveness. Singh et al. [14] developed nanoparticles based on poly(d,l-lactic-co-glycolic acid)(PLGA) with photoprotective properties for the delivery of sparfloxacin, a broad-spectrum antibiotic. Polycaprolactone nanoparticles loaded with doxorubicin were obtained by Heshmatnezhad et al. [15] using a flow-focusing microfluidic device. These polymeric nanoparticles exhibit a sustained drug release profile for up to 10 days, making them a promising platform for chemotherapy applications.

To address the demand for the development of new polyester materials, our group reported the synthesis and characterization of a new polymacrolactone named poly(ethylene brassylate-co-squaric acid) (PEBSA), obtained from renewable sources through the polymerization of ethylene brassylate with squaric acid by two methods, respectively, in solutions and suspension [16,17,18,19]. Polymers and copolymers prepared from ethylene brassylate, a commercially available 17-member macrolactone, have shown the ability to form various supramolecular structures, improved mechanical and thermal properties, and good biodegradation susceptibility compared to other polyester materials [20,21,22]. The use of ethylene brassylate alongside squaric acid, a strong acid with polar functional groups, in a copolymerization synthesis provides an alternative approach for producing materials with tuneable characteristics. PEBSA, with its amphiphilic character, thermo-responsive behaviour, good thermal and mechanical properties, and adequate biocompatibility, emerges as a promising candidate for various applications.

Starting from our previous reports, this study was undertaken to synthesize PEBSA with good water dispersibility by using an emulsion polymerization method. Emulsion polymerization allows the preparation of water-stable latexes consisting of particles with relatively homogeneous sizes and narrow molecular weight distributions in a more environmentally friendly and cost-effective manner. The use of water as a reaction medium improves heat transfer capacity and reduces the viscosity problems encountered in bulk polymerization [22,23].

Norfloxacin (NRF), a second-generation of quinolone synthetic antibiotic, is prescribed in current clinical practice against both Gram-positive and Gram-negative bacterial infections and also against microorganism infections like *Chlamydia pneumoniae* or *Mycoplasma pneumoniae* [24]. This antibiotic is most often administered orally, with its main mechanism of action being the inhibition of the bacterial enzyme DNA gyrase [6]. The main drawbacks of norfloxacin are its low water solubility, reduced half-life time in blood serum and plasma, and low absorption rates.

Considering the accelerated development of multiresistant bacterial species and the large number of deaths caused by infections (nearly 17 million annually) [25], drug delivery systems for specific bioactive structures represent a promising alternative to classic formulations. Numerous studies have been focused on the development of various types of NRF delivery platforms, like hydrogels [26,27], nano and microparticles [5,6,28], or floating microballoons [29]. The primary objective of this research was to enhance NRF oral bioavailability, improve drug solubility and stability, and confer a controlled release.

In this sense, the polymeric microparticles used as norfloxacin delivery systems were designed and synthesised starting from PEBSA copolymer obtained by using the emulsion polymerization method. Three variants of microparticles were prepared using a simple precipitation method with variations in the copolymer/drug ratio. The structure, dimensions, morphology, and release capacity of the microparticles were characterized with particular attention given to the influence of the copolymer/drug ratio. Antimicrobial activity and in vitro biocompatibility were also analysed. The information provided on the synthesis and characterization of microparticles based on PEBSA_50/50__Brij, used for the oral administration of norfloxacin contributes to the expansion of biomedical applications of polymacrolactones and demonstrates their potential for further improvement.

## 2. Materials and Methods

### 2.1. Materials

All the reagents used were of analytical purity and employed without further purification: ethylene brassylate (EB, 1,4-dioxacycloheptadecane-5,17-dione, C_15_H_26_O_4_, Mw = 270.36 g/mol, purity (GC) > 95.0%), squaric acid (SA, 3,4-dihydroxy-3-cyclobutene-1,2-dione, H_2_C_4_O_4_, Mw = 114.06 g/mol, purity (HPLC) > 99.0%), norfloxacin (NRF, 1-ethyl-6-fluoro-1,4-dihydro-4-oxo-7-(1-piperazinyl)-3-quinolinecarboxylic acid, C_16_H_18_ FN_3_O, Mw = 319.33), dimethylsulfoxid (DMSO, (CH_3_)_2_SO, Mw = 78.13 g/mol) and Tween 80 (polyoxyethylenesorbitan monooleate) were purchased from Sigma-Aldrich Co., (Burlington, MA, USA), 1-hexanol anhydrous from Across-Organics (Geel, Belgium), Brij 58 (polyoxyethylene (20) cetylether, HO-(CH_2_CH_2_O)n-(CH_2_)_15_-CH_3_) from Merck (Hohenbrunn, Germany), while hydrochloric acid (HCl, Mw 36.5) was acquired from Chimreactiv (Bucuresti, Romania). The ultrapure water used in the experiments was prepared with a Milli-Q device.

### 2.2. Synthesis of the Copolymer

A new variant of the PEBSA copolymer was synthesised through aqueous emulsion polymerization and coded PEBSA_50/50__Brij. The copolymer, with a 1/1 molar ratio between comonomers, was synthesised through the ring opening of EB, followed by condensation with SA, using 1-hexanol as an initiator and Brij 58 as a surfactant. Summarily, 1.297 mL of EB (5 mmole), 0.57 g of SA (5 mmole), initiator (1 hexanol—0.87 mmole) and surfactant (Brij 58—0.031 mmole) were poured into 20 mL of ultrapure water and steered at 400 rpm. The reaction was performed at 95 °C under a nitrogen atmosphere for 24 h. After the reaction ended, the copolymer was separated by centrifugation, washed several times with water, and freeze-dried for further characterization and use.

### 2.3. Preparation of NRF-Loaded Microparticles

The polymeric microparticles were prepared through a precipitation method, with NRF being incorporated in situ into the polymeric matrix via the inclusion/complexation process. In the first step, 2% *w*/*v* solutions in DMSO were prepared by magnetic stirring at room temperature of predetermined amounts of PEBSA_50/50__Brij and, respectively, NRF. Then, the NRF solution was added dropwise to the copolymer solution at specific ratios (Table 1) under high-speed magnetic stirring at 37 °C. After mixing, the samples were subjected to gentle magnetic stirring for 24 h. Finally, the copolymer/drug solutions were dropped into a water solution of 0.25% Tween80 under vigorous magnetic stirring. The obtained microparticles were separated by centrifugation at 9500 rpm for 15 min.

The purification process involved centrifugation at 9500 rpm for 15 min, washing, and redispersion of the microparticles in distilled water three times. PEBSA-free microparticles, namely PEBSA without drug—(PM_0_), were prepared in similar conditions and used as a reference in further analysis. The analysis in a solid state was conducted on freeze-dried samples. The samples were frozen through immersion in liquid nitrogen in order to be further freeze-dried. Lyophilization was performed using a Martin Christ Apha 2-4 LSCplus (Osterode am Harz, Germany) laboratory freeze dryer at a 0.01 mbar vacuum for 48 h.

### 2.4. Copolymer Characterization

#### 2.4.1. Polymacrolactone Molecular Weight

Gel Permeation Chromatography analysis was performed using the multidetector system WGE SEC-3010 (WGE Dr. Bures GmbH & Co., KG, Dallgow-Doeberitz, Germany) equipped with two PL gel columns (PLgel 5micro Mixed C and PLgel 5micro Mixed D) from Agilent (Santa Clara, CA, USA). The dual refractometer/viscometer detector was calibrated using PS standards with a narrow molecular weight distribution (580–1,350,000 Da). Determination was performed with a 1.0 mL/min dimethylformamide (DMF) flow at 30 °C. Data acquisition and analysis were achieved using ParSEC Chromatography software ver. 5.67.

PEBSA_50/50__Brij presents the following molecular weights: molecular weight of the highest peak (M_p_): 5070 Da; number average molecular weight (M_n_): 4531 Da; weight average molecular weight (M_w_): 4799 Da; higher average molecular weight (M_z_): 5076 Da; higher average molecular weights (M_z+1_): 5432 Da; viscosity average molecular weight (M_v_): 4740 Da; and a polydispersity index (D) of 1.059.

#### 2.4.2. Structural Analyses

Fourier transform infrared spectroscopy (FTIR) analysis of the samples was performed using the Bruker Vertex 70 Spectrometer (Ettlingen, Germany). Briefly, freeze-dried samples were mixed with potassium bromide and compressed into a disk shape for analysis. Spectra were recorded using the transmittance mode in the range 4000–400 cm^−1^ with a 4 cm^−1^ resolution and an average of 64 scans.

^1^H-NMR analysis of the copolymer was accomplished on Bruker Neo Instrument spectrometer from Bruker BioSpin (Rheinstetten, Germany), working at 400.1 MHz. The sample was initially solubilized in DMSO d6 and then transferred to a 5 mm multinuclear inverse detection z-gradient probe. Spectra were recorded at room temperature, with the chemical shifts presented in δ units (ppm) and referenced to the internal deuterated solvent calibrated at 2.512 ppm.

#### 2.4.3. Thermal Analysis

The thermal properties of the copolymer sample were investigated using the TG/FTIR/MS thermal analysis system consisting of an STA 449 F1 Jupiter thermobalance, Netzsch, Selb, Germany, coupled online with a Vertex 70 FTIR spectrometer (Ettlingen, Germany) and with an Aëolos QMS 403C mass spectrometer from Netzsch. Briefly, 14 mg of freeze-dried sample was placed in an open Al_2_O_3_ crucible and heated in a nitrogen-inert atmosphere (50 mL/min gas flow) up to 675 °C. Measurement was achieved in dynamic mode with a heating rate of 10 °C/min using Proteus 5.0.1 software. Gases evolved during the decomposition process were transferred through a heated Teflon line (190 °C) to the TGA-IR external modulus fitted with an MCT (Mercury Cadmium Telluride) detector. FTIR spectra were registered between 4000 and 600 cm^−1^ in 3D size with OPUS 6.5 software. The gases were also transferred to the Aëolos mass spectrometer using a 75 μm quartz capillary maintained at 260 °C. MS spectra ranging from 1 to 200 *m*/*z* were obtained using ionization energy with an electron impact of 70 eV and a vacuum of 10^−5^ mbar. Aëolos 32 software was employed in MS data acquisition and processing.

### 2.5. Characterization of Bioactive Microparticles

#### 2.5.1. Dynamic Light Scattering (DLS) Measurements

The DLS technique was used to determine the mean hydrodynamic diameter (D_h_), polydispersity index (PDI), and zeta potential (ZP) of the synthesised microparticles. Determinations were performed using the Zetasizer Nano ZS device from Malvern Panalytical, Worcestershire, UK. equipped with a 633 nm wavelength red laser (He/Ne). The system uses a non-invasive back scattering technology, NIBST, in order to reduce the multiple scanning effects. Briefly, 2 mL of 0.25% *w*/*v* microparticle solution in water and 2 mL of 2% *w*/*v* NRF solution in DMSO were analysed in the whole measurement range (0.6 nm to 6 μm) using the Mie methods. The hydrodynamic diameter (D_h_) was calculated by using the following Equation (1):

(1) $$D_{h}=\frac{\mathrm{kT}}{3\pi \eta D},$$

where D_h_ is the hydrodynamic diameter, k—Boltzmann constant, T—temperature, η—viscosity, and D—diffusion coefficient.

Zeta potential was determined on the same apparatus using the Smoluchowski relationship (2):

(2) $$\zeta =\frac{\eta \mu }{\varepsilon },$$

with η—viscosity, electrophoretic mobility—noted μ and ε, which represent the dielectric constant of the medium. All the data are expressed as a mean ± standard deviation (SD).

#### 2.5.2. Morphological Characterization

To characterize the morphology and size of the samples, scanning transmission electron microscopy (STEM) was used. This microscopy technique assumes the scanning of the samples with a focused electron beam to produce a transmission image. Micrographs were registered using the Verios G4 UC scanning electron microscope from Thermo Scientific (Brno, Czech Republic) equipped with a STEM 3+ detector. Analyses were performed in bright-field mode and with an accelerating voltage of 30 kV. Microparticles dispersed in water and magnetically stirred, were deposited on 300 mesh carbon-coated copper grids before investigation. The average diameter of polymeric microparticles was determined using ImageJ 1.48v analysing software.

#### 2.5.3. NRF Loading

The NRF encapsulation for PM_1_, PM_2_, and PM_3_ samples, namely the encapsulation efficiency (EE%) and loading capacity (LC%), were calculated using the following equations:

(3) $$EE\%=\frac{\mathrm{initial}\;\mathrm{amount}\;\mathrm{of}\;\mathrm{drug}\text{-}\mathrm{amount}\;\mathrm{of}\;\mathrm{unloaded}\;\mathrm{drug}\;}{\mathrm{initial}\;\mathrm{amount}\;\mathrm{of}\;\mathrm{drug}}\times 100$$

(4) $$LC\%=\frac{\mathrm{amount}\;\mathrm{of}\;\mathrm{drug}\;\mathrm{loaded}\;\mathrm{into}\;\mathrm{microparticle}}{\mathrm{amount}\;\mathrm{of}\;\mathrm{polymer}\;+\;\mathrm{amount}\;\mathrm{of}\;\mathrm{drug}\;\mathrm{loaded}\;\mathrm{into}\;\mathrm{microparticle}}\times 100$$

The amount of free NRF was determined using the spectrophotometric method (Jenway 6305 UV–VIS Spectrophotometer, Stone, Staffordshire, UK) as follows: 1 mL of the supernatant resulting from the centrifugation at 9500 RPM for 15 min of the synthesised microparticle suspension was analysed at 278 nm wavelength.

#### 2.5.4. In Vitro Drug Release Studies

The in vitro NRF release profile was investigated through the spectrophotometric method using the Jenway 6305 UV/Visible spectrophotometer (Stone, Staffordshire, UK). The experiments were carried out using the dialysis bag method in 0.1 M hydrochloric acid medium (HCl) to mimic the in vivo stomach conditions, in accordance with the literature [5,30]. Briefly, 3 mg of microparticles were dispersed in a 0.1 M HCl solution, transferred into a dialysis bag (molecular weight cutoff of 14,000 Da), and immersed in 25 mL of 0.1 M HCl medium. All the experiments were performed at room temperature and under gentle shaking. At different time intervals, specifically: 2, 5, 10, 15, 20, 25, 30, 45, 60, 90, 120, 180, 240, 300, 360 min, and 24 h, 1 mL was withdrawn from the outside of the dialysis bag and analysed at the 278 nm wavelength. Cumulative NRF release was calculated using the equation of a previously constructed calibration curve, with all the data presented as mean ± standard deviation (*n* = 3).

To characterize the release mechanism, the dissolution data (M_t_/M_∞_ < 0.6) were evaluated. Drug release data were fitted using zero-order, first-order, Higuchi (5) [31], and Korsmeyer–Peppas kinetics models (6) [32]. Due to the well fit (R^2^ > 0.9), only the data obtained for the Higuchi and Korsmeyer–Peppas models are listed. The Higuchi model describes the fraction of drug release from a matrix as proportional to the square root of time:

(5) $$M_{t}/M_{\infty }=k_{H}t^{1/2},$$

where M_t_ and M_∞_ are cumulative amounts of drug release at time t and infinite time, and k_H_ is the Higuchi dissolution constant reflection formulation characteristic. If the Higuchi model of drug release (i.e., Fickian diffusion) is obeyed, then a plot of M_t_/M_∞_ versus t^1/2^ will be a straight line with a slope of k_H_.

The Korsmeyer–Peppas model (Power Law) describes the drug release from the polymeric system in which

(6) $$M_{t}/M_{\infty }=kt^{n},$$

(7) $$\log(M_{t}/M_{\infty })=\log k+n\log t$$

where M_t_ and M_∞_ are cumulative amounts of drug release at time t and infinite time, k is a constant that defines the characteristics of a polymer network system, and n is a diffusional release exponent indicative of the mechanism of the drug release for drug dissolution. This equation has two distinct physical and realistic meanings in the two special cases of n = 0.5 (indicating diffusion-controlled drug release) and n = 1 (indicating Case-II release). Between these two limiting cases, the non-Fickian (anomalous or coupled diffusion/relaxation) release behaviour is found in the intermediate between Fickian and Case-II. The release is defined as the non-Fickian when the n value is between 0.5 and 1.0 in the semi-empirical equation. It has been underlined that the two extreme values for the exponent n, 0.5 and 1.0, are only valid for slab geometry. For spheres and cylinders, different values have been derived.

#### 2.5.5. Antimicrobial Activity

The antimicrobial activity of the samples was determined by disk diffusion assay [33,34,35] against *Staphylococcus aureus* ATCC25923, *Escherichia coli* ATCC25922, and *Enterococcus faecalis* ATCC29212. All microorganisms were stored at −80 °C in 20% glycerol. The bacterial strains were refreshed on nutrient agar (NA) at 37 °C. The three cultures were used to prepare microbial suspensions in sterile solution, which presents optical turbidity similar to 0.5 McFarland standards. Volumes of 0.1 mL from each inoculum were spread onto NA plates, and then 15 µL of the samples were added.

To evaluate the antimicrobial properties, the growth inhibition was measured under standard conditions after 24 h of incubation at 37 °C. All tests were carried out in triplicate. After incubation, the samples were analysed with SCAN1200^®^, version 8.6.10.0 (Interscience, Saint-Nom-la-Bretèche, France), and presented as the mean ± standard deviation (SD) using XLSTAT Ecology software, version 2019.4.1.

#### 2.5.6. In Vitro Cytocompatibility

The in vitro cytotoxicity tests were performed using the following reagents suitable for cell culture assays: Dulbecco’s Modified Eagles Medium—DMEM F_12_ HAM; fetal bovine serum—FBS; a mixture of antibiotics (penicillin-streptomycin-neomycin mixture—P/S/N, with approximately 5000 units of penicillin, 5 mg of streptomycin, and 10 mg of neomycin per millilitre, filtered at 0.1 μm); Thiazolyl Blue Tetrazolium Bromide—MTT; Dimethyl Sulfoxide–DMSO. The reagents used in the experiment were obtained from Sigma-Aldrich (Steinheim, Germany).

Primary human dermal fibroblast cells (HDFA cell line) were placed in a complete medium called Dulbecco’s Modified Eagles Medium (DMEM F_12_ HAM). This medium was supplemented with 10% fetal bovine serum (FBS) and a combination of 1% penicillin, streptomycin, and neomycin (P/S/N). The cells were then incubated for 24 h at a temperature of 37 °C with a 5% concentration of carbon dioxide (CO_2_) and a relative humidity of 95%. To conduct the cytocompatibility investigation, 50 µL of cell suspension containing 12 × 10^3^ cells/50 µL cells were seeded in a 96-well plate with 100 µL of complete DMEM-F_12_ HAM added to each well. Subsequently, the cells were incubated for 24 h at 37 °C in a 5% CO_2_ atmosphere to facilitate their attachment to the substrate of the well plates.

Each material underwent sterilization by being subjected to 30 min of UV radiation exposure on both sides. The materials were introduced in contact with cells and subsequently placed in a 5% CO_2_ environment at a temperature of 37 °C, for different periods of time (24 h, 48 h, and 72 h). In addition, the wells containing cells without any materials, which were selected as controls, were replenished with new media to complete the DMEM—F_12_ HAM.

To perform the MTT test, the materials were removed from the wells, and the culturing medium was replaced with the MTT working solution (MTT 5% in unsupplemented DMEM-F_12_ HAM culture medium). Cells in the MTT solution were incubated at 37 °C for 3 h. Formazan was solubilized with DMSO, 150 μL/well. The absorbance of the resulting formazan solution (purple colour) was measured at a wavelength of 570 nm (plate reader, Tecan Sunrise Plate Reader, Männedorf, Switzerland).

The results of the spectrophotometric evaluation of the experimental wells were compared to those of the control wells (no material present). Cell viability was calculated by using

(8) $$\mathrm{Cell}\;\mathrm{viability}=\frac{\mathrm{abs}\;\mathrm{sample}\;}{\mathrm{abs}\;\mathrm{control}}\times 100$$

where the abs sample represents the absorbance of the materials in the well, while abs control is the absorbance of the control.

The MTT test was performed for 24, 48, and 72 h, respectively, in triplicate. To sustain MTT results, cell morphology and density were studied using an Inverted Phase Contrast Microscope (Leica, Wetzlar, Germany) and images were taken (10× objective).

To highlight the morphology of the cells, live/dead staining was involved. HDFA cells were incubated with samples in the same condition described above, in 48-well plates (15 × 10^3^ cells/well), for 72 h.

The live/dead staining experiment was conducted to observe the cells, employing a Calcein AM dye solution. The dye solution, with a concentration of 2 µL/mL, was introduced into the cell cultures and allowed to incubate for 30 min at a temperature of 37 °C.

Images were captured using a 10× objective from a Leica DM IL LED Inverted Microscope with a Phase Contrast System and Fluorescence (Leica Microsystems GmbH, Wetzlar, Germany).

### 2.6. Statistical Analysis

All data in this study were presented as mean ± standard deviation (*n* = 3) with error bars. Statistical analysis was carried out by a one-way or two-way analysis of variance (ANOVA) test followed by Tukey’s post hoc test among multiple groups or Student’s *t*-test between two groups using GraphPad Prism software (GraphPad Prism 7.00). At a probability value of *p* < 0.05, the data were considered to be statistically significant, and the significance is noted as * *p* < 0.05; ** *p* < 0.01; *** *p* < 0.001; and **** *p* < 0.0001.

## 3. Results and Discussion

### 3.1. Characterization of the Copolymer

#### 3.1.1. Structural Characterization

**Figure 1:**
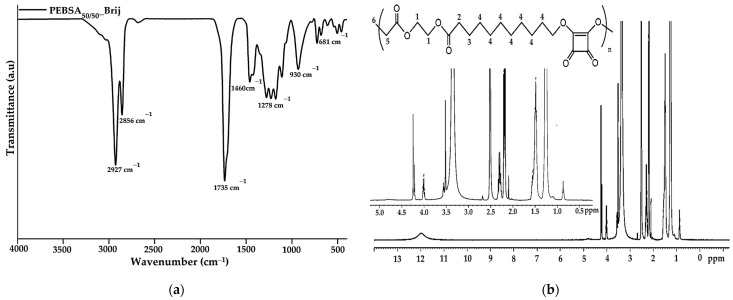
FTIR (**a**) and ^1^H-NMR (**b**) spectra of copolymacrolactone.

FTIR characterization was employed to analyse and confirm the structure of the obtained copolymer. The spectrum of PEBSA_50/50__Brij (Figure 1a) illustrates the characteristic peaks of PEBSA copolymer previously reported in the literature without significant changes [17,18,19]. Therefore, the success of the copolymer synthesis is confirmed by the disappearance of the bands specific to the hydroxyl group of squaric acid as a result of its condensation reaction with the open lactone cycle of ethylene brassylate. The characteristic PEBSA_50/50__Brij peaks are assigned to the stretching vibration of carbonyl C=O bonds (1735 cm^−1^) and asymmetric, symmetric, and bending vibrations of C-H bonds (2927, 2856, and 1460 cm^−1^). Other distinguishing bands are at 1177 cm^−1^ corresponding to C-O-C stretching vibrations, 1278 and 1228 cm^−1^ of C-O stretching vibrations, and C=C bending vibration at 930 and 681 cm^−1^.

The ^1^H-NMR spectra of the synthesized copolymacrolactone illustrated in Figure 1b support the FTIR data, confirming the chemical structure. The spectra present characteristic signals assigned to PEBSA_50/50__Brij at: 11.98 (s, OH), 4.00 (q, CH_2_—1), 2.19 (t, CH_2_—2), 1.50 (m, CH_2_—3), 1.25 (s, CH_2_—4), and 0.87 (t, CH_3_—6), these corresponding to the notation of the protons identified in the structure. The synthesis is confirmed by the presence of end group-specific signals: 11.98 ppm of OH from squaric acid units and 0.87 ppm of methylene protons (6), as well as by the chemical shifts of methylene protons in the EB structure (1, 2, 3, 4). All these signals are in good agreement with the literature data regarding the PEBSA copolymer previously reported, supporting the similar chemical structure of the copolymer synthesised through different methods [17,18,19]. In addition, the signals observed at 3.55, 3.49, 3.35, and 2.09 ppm are attributed to protons characteristic of residual Brij 58 fragments, which are responsible for the good colloidal stability of the polymeric microparticles.

#### 3.1.2. Thermal Characterization

**Figure 2:**
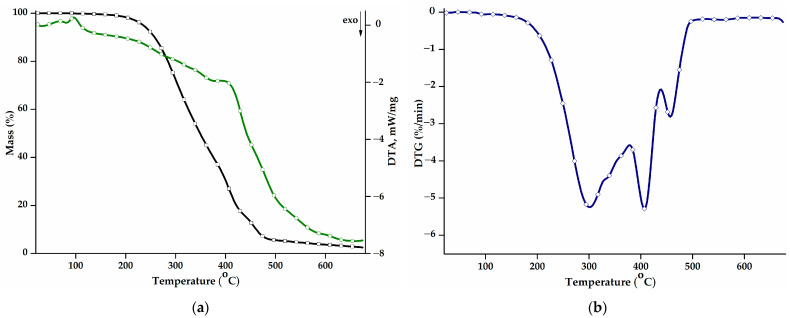
(**a**) Mass loss (TG, black) and differential thermal analysis (DTA, green) curves, and (**b**) DTG curve corresponding to the PEBSA_50/50__Brij copolymer.

The thermal behaviour of the PEBSA_50/50__Brij copolymer was studied in the range of 30–675 °C. Figure 2a shows the mass loss (TG curve) and the differential thermal analysis curve (DTA) as a function of the temperature, while Figure 2b presents the first derivative of the TG curve, DTG. Additional information on the values of the main thermal parameters is displayed in Table 2. The thermal decomposition process of PEBSA_50/50__Brij presents three stages and a residual mass of 2.50% up to 675 °C, consistent with the thermal analyses of the copolymer synthesised by different polymerization methods and previously reported [16,17,18].

The main decomposition stage illustrated by the DTG curve is the first stage and exhibits a T_peak_ at 301 °C, a shoulder at 338 °C, and a total mass loss of 61.15%. During this stage, squaric acid sequences decompose into low-molecular-weight volatile compounds like water, carbon monoxide, carbon dioxide, and hydrocarbons [36]. The second stage begins at 386 °C and reaches its maximum degradation rate at 408 °C. The mass loss registered during this stage can be attributed to the further degradation of the squaric acid units and also to the onset of the degradation of macrolactone units.

The last degradation stage recorded at high temperatures, above 442 °C, corresponds to the degradation of ethylene brassylate fragments. The breaking of carbon-carbon bonds results in the release of various products, such as saturated and unsaturated aliphatic and cyclic hydrocarbons, aromatic hydrocarbons, and carbonyl compounds, thus producing a mass loss of 13.32% [20].

The thermal stability of the polymacrolactone was determined according to the values of the T_10_ and T_20_ parameters. Compared to the copolymer obtained through polymerization in solution (T_10_: 287 °C; T_20_: 310 °C) and suspension (T_10_: 268 °C; T_20_: 283 °C) [18], the PEBSA_50/50__Brij variant presents a small decrease in terms of thermal stability and decomposition temperatures. The values of the DTA parameter exhibit the same trend, registering slightly decreased values, compared to the other PEBSA variants. Polymers obtained through emulsion polymerization generally exhibit lower thermal properties due to a higher molecular weight polydispersity index, the presence of residual surfactant molecules, or a decrease in the crystallization degree [37,38].

**Figure 3:**
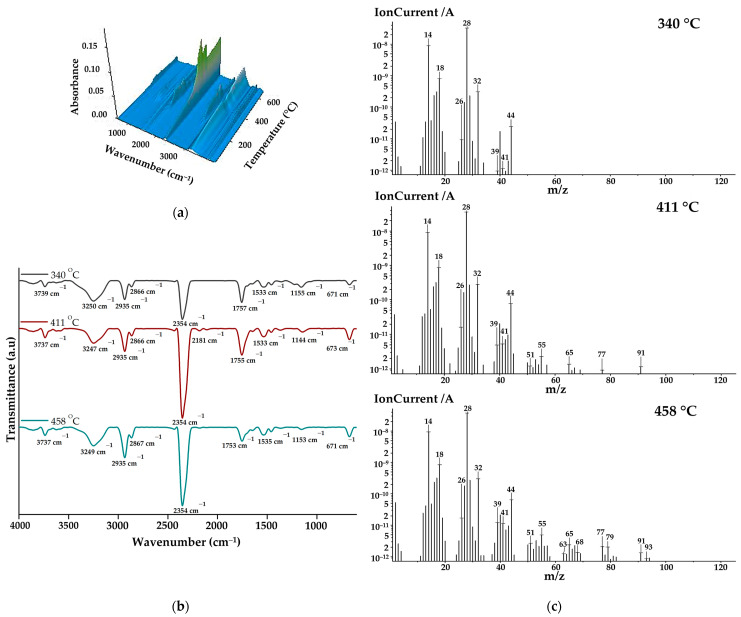
(**a**) 3D FTIR spectra, (**b**) 2D FTIR spectra, and (**c**) MS spectra of degradation products.

The gases that evolved during the thermal decomposition of copolymacrolactone were analysed through FTIR and mass spectroscopy employed concomitantly with thermogravimetric measurement using the TG/FTIR/MS system. Figure 3a illustrates the 3D FTIR spectrum of PEBSA_50/50__Brij main released decomposition products. From the 3D spectra, the 2D spectra were extracted at temperatures corresponding to the maximum amount of gases, near the DTG peaks (Figure 3b). As can be seen from the FTIR spectra, the main decomposition products are released within the 225–480 °C temperature range. The most important bands identified in the spectra were O=C=O stretching vibration at 2354 cm^−1^, C-H asymmetric at 2935 cm^−1^, and symmetric stretching vibration at 2866 cm^−1^, C=O stretching vibration at ~1755 cm^−1^, and O-H stretching vibration between 3855 and 3621 cm^−1^. Other registered absorption bands correspond to O-H bending vibration at ~1367, C-O stretching vibration at 1223 cm^−1^, C-O-C bridge stretching vibration at ~1155 cm^−1^, and C=C stretching vibration at 1647 cm^−1^ and 1533 cm^−1^. Under 1000 cm^−1^, O=C=O bending vibration can be observed at 671 cm^−1^. All spectra exhibit the MCT detector-specific “ice band” at ~3249 cm^−1^ [39]. Starting from the spectral assignments made, the main volatile compounds evolved during copolymer decomposition are carbon dioxide (2354 and 671 cm^−1^), aliphatic and cyclic saturated hydrocarbons (2935, 2866, and 1458 cm^−1^) water and alcohols (bands from 3855–3621, 1367, and 1223 cm^−1^) aromatic and unsaturated hydrocarbons (1647 and 1533 cm^−1^), and aliphatic carbonyl derivatives (1755, 1233, and 1155 cm^−1^). The increased intensities of carbonyl and hydroxyl groups observed in the spectra registered at 340 and 411 ^o^C indicate that alcohols, water, and carbonyl derivates like aldehyde or ketones are primarily released during the first and second degradation stages. As the temperature increases, the intensities of hydrocarbon-specific bands grow gradually due to ethylene brassylate moiety decomposition.

The MS spectra of the main volatile compounds evolved during the decomposition of PEBSA_50/50__Brij are illustrated in Figure 3c. As can be seen, at 340 °C, most of the signals are located below 50 *m*/*z,* while at 410 °C and 445 °C, signals can also be observed at higher *m*/*z* values. This can be correlated with the fact that at lower temperatures, the breakdown of squaric acid units produces low-molecular-weight oxygen-containing products and hydrocarbons. When the temperature rises above 400 °C, aliphatic and aromatic hydrocarbons with higher molecular weights are released, especially due to macrolactone degradation. The assignments of the main volatile compounds from the MS spectra were performed in accordance with the literature [16,17,18,39,40] and the NIST spectral data base [41]. The most significant ionic fragments identified in the spectra can be assigned to: carbon dioxide (CO_2_: 44, 28, 16 *m*/*z*), carbon monoxide (CO: 28, 29 *m*/*z*), water (H_2_O: 18, 17 *m*/*z*), methanol (CH_4_O: 31, 32, 30 *m*/*z*), ethanol (C_2_H_6_O: 31, 45, 29, 27 *m*/*z*), 2-propenal (C_3_H_4_O: 56, 27, 26, 55 *m*/*z*), acetaldehyde (C_2_H_4_O: 29, 44, 43, 15 *m*/*z*), methane (CH_4_: 16, 15, 14 *m*/*z*), cyclopropane (C_3_H_6_: 42, 41, 39, 40 *m*/*z*), cyclopropene (C_3_H_4_: 39, 40, 38, 37 *m*/*z*), cyclobutene (C_4_H_6_: 39, 54, 53, 27, 50 *m*/*z*), 1,3-pentadiene (C_5_H_8_: 67, 68, 53, 65 *m*/*z*), benzene (C_6_H_6_: 78, 77, 51, 50, 79 *m*/*z*), and toluene (C_7_H_8_: 91, 65, 63, 93 *m*/*z*). All presented MS data align with previous studies on PEBSA’s thermal decomposition and agree with FTIR assignments [16,17,18].

### 3.2. Characterization of Bioactive Microparticles

#### 3.2.1. FTIR Characterization

**Figure 4:**
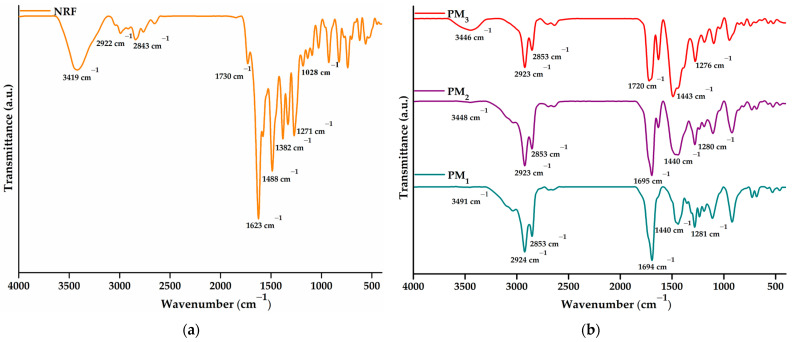
FTIR spectra of (**a**) NRF and (**b**) microparticles.

The successful incorporation of norfloxacin into the obtained microparticles and the drug-polymer interactions were investigated using FTIR spectroscopy. Figure 4a presents the spectra of NRF, whereas Figure 4b depicts the spectra of PM_1_, PM_2_, and PM_3_ samples, respectively.

The NRF spectrum presents bands specific to O-H at 3419 cm^−1^ (stretching vibration) and 1271 cm^−1^ (bending vibration) from the hydroxyl functional groups and N-H of the imino moiety of piperazinyl groups at 3301 cm^−1^ (stretching vibration) and 1623 cm^−1^ (bending vibration) [42]. Carboxylic groups of norfloxacin exhibit specific bands at 1730 cm^−1^ due to C=O stretching vibration, 1488 cm^−1^ assigned to O-H bending vibration, and 1382 cm^−1^ corresponding to C-O bending vibrations. In addition, the 1028 cm^−1^ band can be assigned to C-F stretching vibration, while 2922 and 2843 cm^−1^ to C-H bond asymmetric and symmetric vibrations.

The FTIR spectrum of the polymeric microparticles loaded with NRF presents bands specific to both constituents, demonstrating the successful encapsulation of NRF in the polymeric structure. The most intense band observed in the spectra is assigned to carbonyl stretching vibration at ~1695 cm^−1^. Bands observed in the region of 3600–3300 cm^−1^ correspond to hydroxyl and amido groups specific to norfloxacin moieties. In addition, the presence of bands at ~1620 and ~1028 cm^−1^ attested to the incorporation of drug molecules into microstructures. Other characteristic bands correspond to C-H asymmetric (2923 cm^−1^) and symmetric vibration (2853 cm^−1^). Hydroxyl bending vibration appears at ~1440 cm^−1^. Several modifications in the spectral features of the microparticles are observed, especially at the bands in the regions of 3600–3300 cm^−1^ and 1800–1300 cm^−1^ from C=O, N-H, and O-H vibrations. The shift of the absorbance values specific to the hydroxyl, carbonyl, and amino groups and the decrease in the intensity of the bands demonstrate their involvement in the formation of intermolecular bonds, especially hydrogen bonds [42,43]. Thus, the encapsulation of a larger amount of NRF leads to an increase in the density of the intermolecular physical bonds that are established between the copolymer and the drug. The above statement is supported by the displacement of the O-H bands to lower values, from 3491 cm^−1^ in the case of PM_1_ to 3446 cm^−1^ for PM_3_.

#### 3.2.2. DLS Investigations

**Figure 5:**
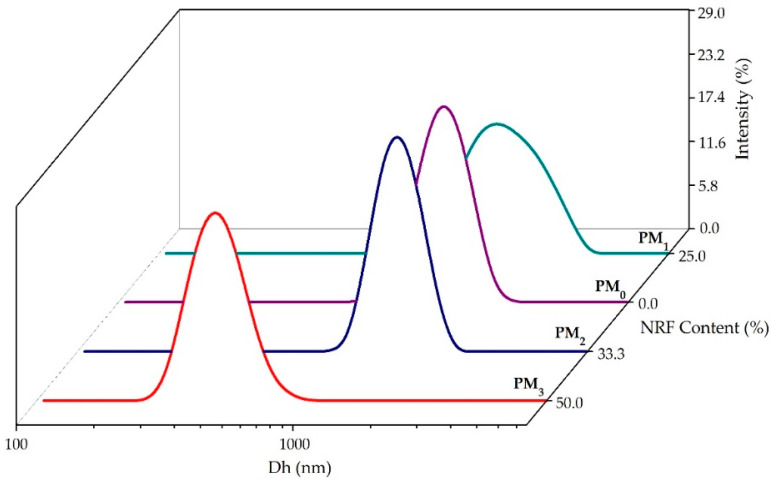
Size distribution of the obtained microparticles.

To gain a deeper understanding of the behaviour of polymeric microparticles in an aqueous state, non-invasive DLS investigations were employed. Figure 5 illustrates the size distribution of polymeric microparticles. Table 3 presents the main DLS parameters, namely hydrodynamic diameter (D_h_), polydispersity index (PDI), and zeta potential (ZP) of the samples.

All the investigated samples presented a monomodal size distribution and hydrodynamic diameter between 429 ± 16 nm for PM_3,_ and 2001 ± 67 nm in the case of the PM_1_ sample (Table 3).

The size reduction of PM_2_ and PM_3_ samples attests to the entrapment of the model drug in the polymer structure and the formation of intermolecular bonds. Increasing NRF content leads to the formation of small drug-loaded microparticles (PM_3_ < PM_2_ < PM_1_), which can be attributed to increased interactions between PEBSA_50/50__Brij and NRF. These interactions ensure better compression inside the particles, resulting in smaller particle sizes. In addition, increasing the amount of copolymer leads to higher viscosity, which promotes chain association and hinders the diffusion of the organic phase into the aqueous phase, resulting in larger microparticles [27,44,45]. Recorded PDI values support the previous statement. Thus, the PDI decreased with the increase in the amount of NRF, being the lowest at the stoichiometric (gravimetric) ratio. In addition, all PDI values are below 0.7, indicating good homogeneity of the microparticles, which also attests to the effectiveness of the surfactant used.

Furthermore, ZP values indicate good colloidal stability of the synthesised microparticles (Table 3). The encapsulation of drug molecules inside the microstructures is underlined by the decrease of the zeta potential from −1.3 mV, corresponding to NRF, to −19 mV for PM_1_. The small difference between the ZP of the microparticles with and without NRF indicates the formation of core–shell-like structures where the surface charge is primarily given by the polymeric shell. This statement is corroborated by the STEM characterization of the microparticles in the following section.

#### 3.2.3. Morphological Characterization

**Figure 6:**
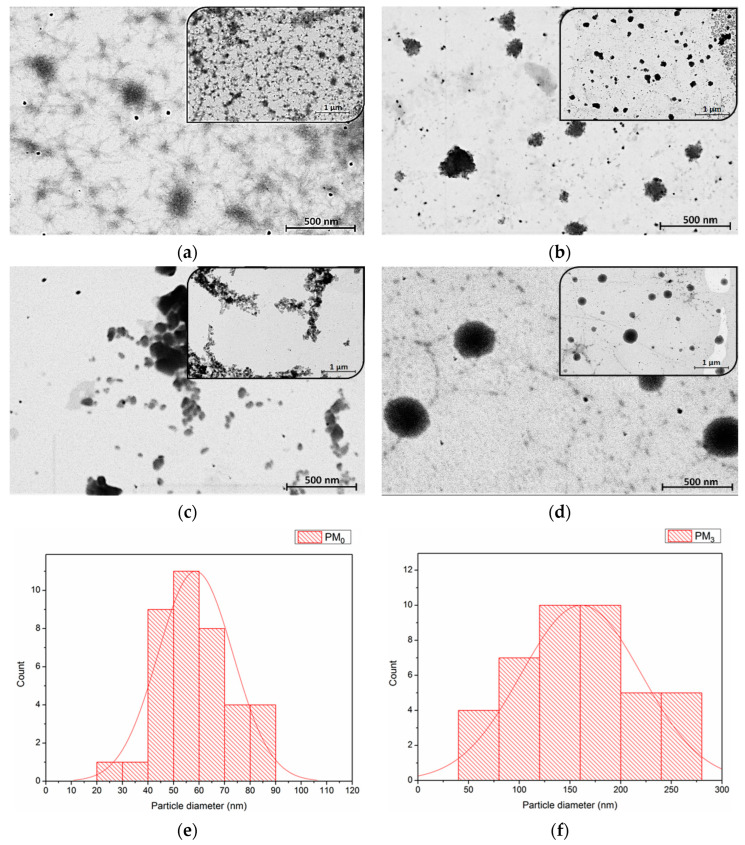
STEM images of microparticles at two magnifications: (**a**) PM_0_, (**b**) PM_1_, (**c**) PM_2_, (**d**) PM_3,_ and particle size distribution histogram from STEM micrographs of PM_0_ (**e**) and PM_3_ (**f**).

STEM analysis was employed to investigate the morphology and size of microparticles in the dry state, as well as the influence of constituent ratios. Figure 6 presents the STEM micrographs of the synthesised microparticles at 20,000× and 50,000× magnifications.

STEM microscopy reaffirms the suitability of PEBSA microparticles (PM_0_) as drug delivery systems [46,47]. As Figure 6b–d presents, all the synthesised bioactive microparticle samples have a roughly spherical shape, with PM_3_ having the most uniform morphology. This fact emphasizes that the incorporation of NRF leads to microstructures with more regular spherical shapes as compared to the pristine PEBSA microparticles due to the copolymer-drug intermolecular interactions. As the copolymer/drug ratio decreases, microparticles with a more regular shape and heterogeneity are obtained as a result of the supplementary physical bonds established. The core–shell structure of the obtained microparticles is evidenced in Figure 6d by the presence of a darker core covered by lighter areas that correspond to the copolymer [48]. To determine the average diameter of the unloaded and loaded microparticles, a total of 38 microparticles were measured using ImageJ 1.48v software. Size distribution histograms of PM_0_ and PM_3_ mounted using Sturges’ criterion (minimum 6 bins in the histogram to visualize this distribution of values) [35,49] are illustrated in Figure 6e,f. The histograms were modelled by the log-normal distribution as presented in the figure. The measurements revealed that pristine microparticles, PM_0_, have an average diameter of 59 ± 1 nm and PM_3_ microparticles have a diameter of 157 ± 2 nm. The dimensional differences that appear between STEM and DLS analysis can be attributed to the fact that microparticles are in a dry, compact state in the first case and a hydrated state in the second one. In the DLS investigation, solvent molecules are attached to the surface of the microparticles through various interactions, which increases the recorded D_h_ [50,51].

#### 3.2.4. Norfloxacin Loading and In Vitro Release Studies

The entrapment efficiency and loading capacity of norfloxacin in the polymeric microparticles are shown in Table 4. The highest values of EE% and LC% are registered for the PM_3_ sample, where an equal ratio of copolymer and drug was used. ANOVA analysis shows a significant increase in the LC% (*p* < 0.001) with a decrease in the copolymer/drug ratio, which can be explained by the high affinity of PEBSA copolymers to incorporate hydrophobic compounds [19,52]. As Table 4 shows, EE% is little influenced by the copolymer/drug ratio, with no significant variations occurring between samples (*p* > 0.05). The use of a higher amount of antibiotic during particle preparation allows for the establishment of a greater number of intermolecular physical bonds and easier penetration of the copolymer matrix. The main physical bonds established between PEBSA_50/50__Brij and NRF are hydrogen bonds exhibited between carbonyl, hydroxyl, and amino functional groups presented on both structures, dipole–dipole interactions manifested among polar covalent bonds like C-F, C=O or C-OH, and also London dispersion forces. The formation of these intermolecular bonds ensures the encapsulation of NRF molecules and controls their release profile.

Norfloxacin is an antibiotic with a pH-dependent solubility that presents four protonated forms. Its zwitterionic form, which is dominant between pH 6.2 and 8, exhibits the lowest solubility [53,54]. Due to the fact that its solubility increases at acidic and, respectively, basic pHs, its primary absorption will occur in the upper compartments of the gastrointestinal tract [55]. Therefore, the study evaluates its release capacity from the synthesised bioactive system in simulated gastric fluid (0.1 M HCl medium).

**Figure 7:**
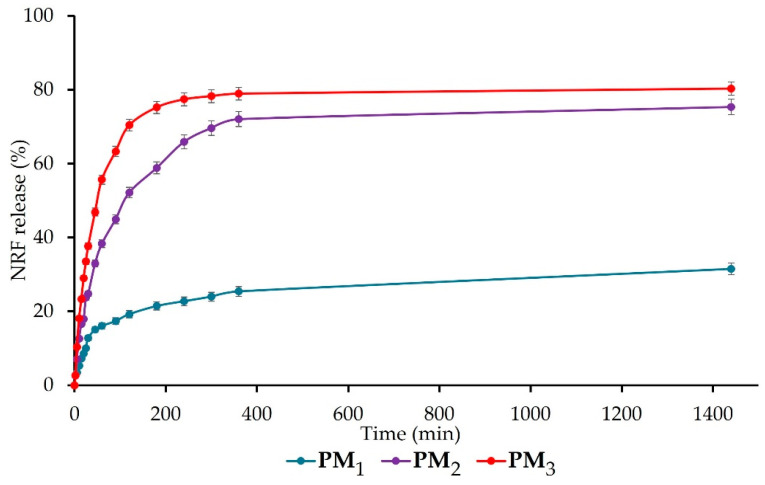
Cumulative drug release of NRF-loaded microparticles.

The release of NRF from the synthesised polymeric microparticles is presented in Figure 7. All samples present a biphasic release profile with a fast (burst) phase up to 120 min, followed by a sustained phase. The burst phase can be assigned to the rapid release of NRF molecules entrapped closer to the surface of microparticles and the high surface-to-volume ratio specific to nano-/microstructures [5]. The percentage of the drug release at the end of the first stage is 19 ± 1% for PM_1_, 52 ± 2% for PM_2_, respectively, 70 ± 2% for PM_3_. The results of the ANOVA test (*p* < 0.001) indicate that the copolymer/drug ratio significantly influences the release rate after 120 min, increasing as the ratio decreases. In addition, the acidic pH of the simulated medium causes the protonation of the drug molecules, which leads to the reduction of physical bonds with the polymer and implicitly to their faster release [42].

After 24 h, the same trend was observed, with the release of NRF from samples registering the following values: PM_1_ = 32 ± 2%, PM_2_ = 75 ± 2%, and PM_3_ = 80 ± 2% (*p* < 0.05 between the three groups). The significant difference that appears between the release profiles of the three microparticle variants is mainly due to the larger number of drug molecules present in the PM_3_ sample, which determines their faster release compared to the PM_1_ sample, which is also correlated with microparticle sizes. This biphasic release behaviour in the simulated gastric fluid is desired for drugs that exhibit narrow absorption windows due to instability or poor solubility in the lower intestine or colon, like norfloxacin [29]. All the obtained data are in agreement with the literature [5,29] and demonstrate the ability of microparticles to ensure a sustained release of NRF into the gastric medium, thus improving bioavailability, absorption rate, and patient compliance.

**Figure 8:**
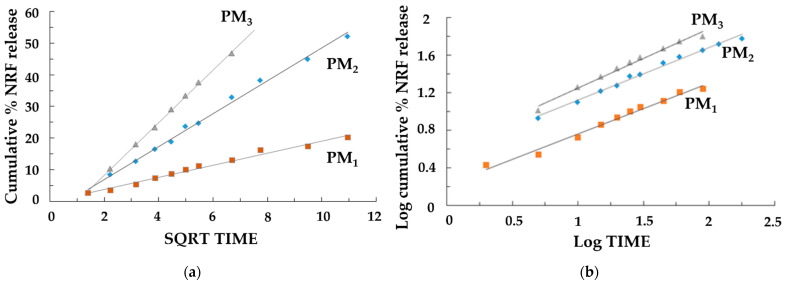
Release kinetics graphs: (**a**) Higuchi model and (**b**) Korsmeyer–Peppas models.

The release of the drug was a controlled release process, mainly by diffusion, because the polymer matrix did not degrade significantly during the experiments. When the norfloxacin dissolution profiles were fitted to different kinetic models, better adjustment was obtained for the Higuchi (Figure 8a) and Korsmeyer–Peppas (Figure 8b) equations, which was indicative, along with the n values (Table 5) of the possible presence of a release mechanism more complex than Fickian diffusion.

The evaluation of the rate constants k_H_ and k’ (Table 5) confirmed that the concentration of NRF directly affects the dissolution rate of the samples. The results showed a higher dissolution rate for the samples where the concentration of NRF was higher (PM_2_ and PM_3_), leading to a rapid release of NRF within the first few minutes. This is due to the greater amount of drug present on the surface of the nanoparticles. Furthermore, the study confirmed that when the copolymer/drug ratio was 3:1, the drug release was slower. In this case, the drug’s effective diffusivity is affected by complexation, causing a slower stage of release after swelling that is characterized by a lower diffusivity of the drug. It should also be emphasized that in this case, the good fit to the Higuchi equation and the n values of 0.508 in the Korsmeyer–Peppas equation indicated a drug release mechanism mainly controlled by Fickian diffusion. For the other samples, the possible presence of a release mechanism more complex than Fickian diffusion occurs, where Fickian diffusion is coupled with the relaxation of the polymer network. It can be concluded that the PM_3_ sample is the most suitable system for its development into new therapeutic formulations with high efficacy for the rapid release of poorly soluble NRF in the treatment of diseases that require rapid drug action.

#### 3.2.5. Antimicrobial Activity

**Figure 9:**
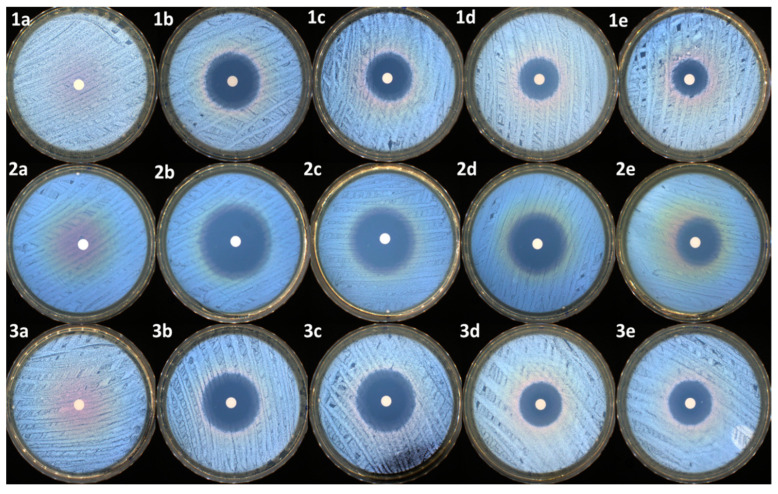
Antimicrobial activity of samples against *S. aureus*: (**1a**)—control, (**1b**)—NRF, (**1c**)—PM_3_, (**1d**)—PM_2_, and (**1e**)—PM_1_; *E. coli*: (**2a**)—control, (**2b**)—NRF, (**2c**)—PM_3_, (**2d**)—PM_2_, and (**2e**)—PM_1_; *E. faecalis*: (**3a**)—control, (**3b**)—NRF, (**3c**)—PM_3_, (**3d**)—PM_2_, and (**3e**)—PM_1_.

As presented in Table 6 and Figure 9, all the samples, except the control (PEBSA_50/50__Brij), present antibacterial activity against the tested reference strains. As expected, the highest antibacterial activity was noticed for the antibiotic sample (NRF) (up to 35 mm of inhibition zone in the case of *E. coli*).

The same activity was maintained in the case of the PM_3_ sample, which has an identical concentration of drug to that of the NRF sample. In the case of PM_2_ and PM_1,_ the reduction of the inhibition zones is correlated with the decrease in the antibiotic dosage. Overall, the samples were very efficient, mostly against the Gram-negative bacterial strain represented by *E. coli*. The same samples were moderated efficiently against the other two Gram-positive bacterial strains, represented by *S. aureus* and *E. faecalis*. *t*-tests were carried out to determine whether the halos produced by the samples were significantly different from the controls (NRF). The results were highly significantly different from the controls in most of the cases (as presented in Table 6), with the antibacterial activity being determined by the NRF concentration within the microparticles.

#### 3.2.6. In Vitro Cytocompatibility

**Figure 10:**
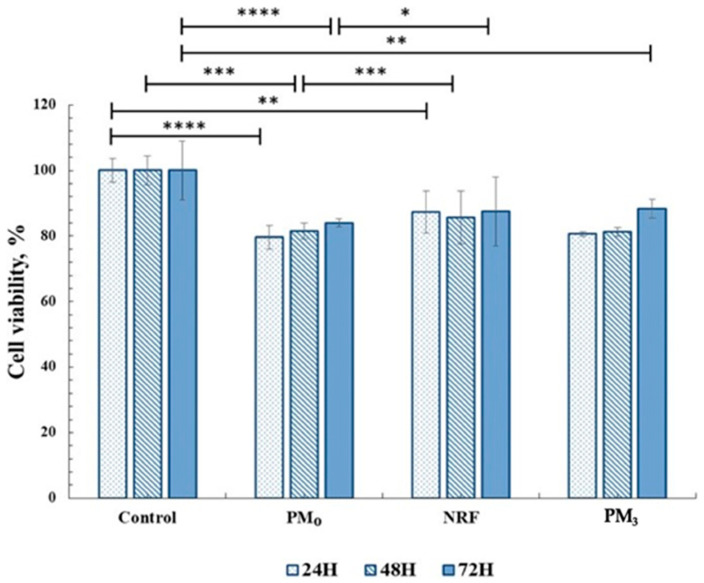
Cell viability data from the MTT assays for all materials tested on fibroblasts (HDFA cell line). Significance levels obtained from two-way ANOVA tests and Tukey’s post hoc analysis are denoted by stars (*), * *p* < 0.05; ** *p* < 0.01; *** *p* < 0.001; and **** *p* < 0.0001.

In order to be used in medical applications as therapeutic systems, any material must be biocompatible, and cytotoxicity tests are a standard method of evaluation [56]. In this regard, the MTT assay was used to highlight the cells response to the PM_3_ microparticles based on PEBSA_50/50__Brij and norfloxacin. The tests were performed on human fibroblast cells. The in vitro experiments were carried out in accordance with the ISO10993-5 standard test method (direct contact) [57]. The results are presented in Figure 10.

Cell viability data for drug-free microparticles, PM_0_, registered values of 83%, and for NRF, 87%, and remain within the cytocompatibility range. After 72 h of incubation, it was observed that the PM_3_ also showed cell viability values over 85%, which demonstrates the compatibility of the system with the cellular environment. This behaviour highlights the fact that drug incorporation, at the tested concentration, preserves the complex interaction with cells.

**Figure 11:**
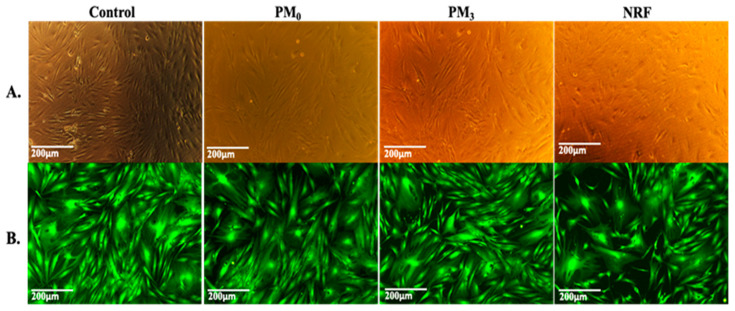
Viable cells and fixed cells, respectively, after 72 h of cell culture with PM_0_, NRF, and PM_3_. Images were achieved through phase contrast microscopy (**A**) and fluorescence microscopy (**B**), captured using a 10× objective.

The MTT data are supported by the morphological test findings, which are presented in Figure 11. The cells exhibit the typical shape of healthy fibroblasts, are attached to the substrate, create a homogenous monolayer, and are morphologically normal [58]. Fluorescence microscope images of representative fields of cells that were in contact with PM_0_, NRF, and PM_3_ cells that were treated with Calcein-AM [59] (Figure 11B). Calcein AM stains only live cells and generates green fluorescence signals. Through this assay, the cells were shown to have a normal shape, forming a confluent monolayer. Human fibroblasts, the HDFA cell line [60], have elongated shapes and grow attached to a substrate, proving to multiply rapidly in cell culture. The ability of the cells to multiply and communicate with each other during the experiment is proof that the PM_3_ microparticles proposed in this study are suitable for tissue engineering.

## 4. Conclusions

Polymeric microparticles designed for the oral delivery of NRF were prepared using PEBSA_50/50__Brij, a copolymer obtained through the emulsion polymerization technique. The polymacrolactone molecular weight, thermal behaviour, and structure were investigated. FTIR analysis confirmed the encapsulation of NRF into polymeric microparticles and highlighted the formation of new physical bonds that occurred between constituents. The DLS investigation revealed that the microparticles have dimensions in the range of 400–2000 nm, which increase proportionally with the copolymer/drug ratio. Zeta potential determinations confirmed the encapsulation of drug molecules inside the PEBSA_50/50__Brij copolymer matrix and the formation of core–shell-like structures. Microparticles have a roughly spherical shape and a relatively homogenous distribution, according to STEM micrographs. All microparticles exhibited good EE% and LC% of NRF and a biphasic drug release profile, with sample PM_3_ presenting the highest release rate of 80 ± 2%.

The antibacterial activity of the samples was also investigated, with the results demonstrating that microparticles are very effective against Gram-negative strains. In addition, PEBSA_50/50__Brij did not inhibit the native antibacterial activity of NRF. Microparticle in vitro biocompatibility was proven by the MTT cytotoxicity assay results against the HDFA cell line.

## Figures and Tables

**Table 1:** Sample code of microparticles as a function of the ratio between constituents.

Samples Code	PEBSA_50/50__Brij/NRF *v*/*v*	Nominal Percentage Composition of a Sample	
		PEBSA_50/50__Brij (%)	NRF (%)
PM_1_	3:1	75%	25%
PM_2_	2:1	66.66%	33.33%
PM_3_	1:1	50%	50%

**Table 2:** Main degradation parameters depicted for PEBSA_50/50__Brij.

Degradation Stage	T_onset_ (°C)	T_peak_ (°C)	W (%)	Residual Mass (%)	T_10_ (°C)	T_20_ (°C)	T_g_ (°C)	T_m_ (°C)	ΔH (J/g)	ΔCp (J/g·K)	T_GS_ (°C)
I	225 ± 2	301 ± 2	61.15 ± 0.01								340 ± 2
II	386 ± 2	408 ± 2	23.03 ± 0.01	2.50 ± 0.01	259 ± 2	285 ± 2	55 ± 2	96 ± 2	37 ± 1	0.33 ± 0.01	411 ± 2
III	442 ± 2	457 ± 2	13.32 ± 0.01								458 ± 2

T_onset_: temperature at which the thermal decomposition begins. T_peak_: temperature corresponding to the maximum decomposition rate; W: mass loss; T_10_, T_20_: temperature of 10% and 20% mass losses; T_g_: glass transition temperature; T_m_: melting temperature; ΔH: variation of enthalpy; ΔCp: variation of heat capacity; T_GS_: the temperature at which the amount of gases evolved is maximum (peaks of the Gram–Schmidt curve).

**Table 3:** DLS parameter values determined for samples.

Samples	D_h_ (nm)	PDI	ZP (mV)
PM_0_	1773 ± 35	0.6 ± 0.1	−17.2 ± 0.6
PM_1_	2001 ± 67	0.31 ± 0.04	−19.0 ± 0.4
PM_2_	1438 ± 8	0.21 ± 0.06	−14.0 ± 0.2
PM_3_	429 ± 16	0.19 ± 0.06	−16.7 ± 0.8
NRF	927 ± 37	0.67 ± 0.09	−1.3 ± 0.5

**Table 4:** Entrapment efficiency and loading capacity of microparticles.

Sample Code	PEBSA_50/50__Brij/NRF *v*/*v*	EE%	LC%
PM_1_	3:1	65 ± 5	18 ± 1
PM_2_	2:1	67 ± 1	25.2 ± 0.3
PM_3_	1:1	69 ± 1	40.9 ± 0.4

**Table 5:** Drug release kinetic parameters.

Samples	Higuchi Equation		Korsmeyer–Peppas		
	k_H_ (min^−0.5^)	R^2^_adj_	n	k’ (min^−n^)	R^2^_adj_
PM_1_	1.92	0.985	0.508	1.3	0.981
PM_2_	4.815	0.991	0.558	4.95	0.991
PM_3_	8.322	0.999	0.673	1.676	0.994

k_h_: Higuchi release rate constant, R^2^_adj_: adjusted correlation coefficient, n: diffusional release exponent, k’: Korsmeyer–Peppas release rate constant.

**Table 6:** Antimicrobial activity of the tested compounds against the reference strains (mm). Significance levels obtained from *t* tests are denoted by stars (*), ** *p* < 0.01; *** *p* < 0.001.

Samples	Antimicrobial Activity (mm)		
	*S. aureus*	*E. coli*	*E. faecalis*
Control	-	-	-
NRF	24.3 ± 0.8	34.8 ± 0.6	34.8 ± 0.6
PM_3_	21 ± 2 **	35 ± 2	22 ± 2 **
PM_2_	13 ± 3 ***	25.1 ± 0.7 **	12.1 ± 0.4 ***
PM_1_	11 ± 2 ***	17.6 ± 0.9 **	11.8 ± 0.3 ***

## Data Availability

The data presented in this study are available in this article.
